# Antiseptic Atomiser

**Published:** 1878-11

**Authors:** 


DR. DE BEER'S *Antiseptic Atomiser* has recently been introduced in Buffalo, together with his Croup Bottle and Medicated Steam Bath. The Atomiser was used in recent clinic with highly satisfactory effect, the instrument throwing a continuous spray, and affording ample opportunity in point of time and extent of surface covered, for any surgical operation. The medicated bath apparatus is admirable for purpose designed. Dr. De Beer in a recent visit to Buffalo, kindly exhibited his various inventions to the profession, and received numerous expressions of appreciation and approval. Mercurial and Sulphur Baths can be readily applied at the bed-side or physicians' office, and his various instruments we have no doubt, will prove a valuable means of applying local medication.

